# Factors Associated with Acute Colonic Pseudo-Obstruction After Cesarean Section: A Systematic Review and Meta-Analysis

**DOI:** 10.3390/jcm15082817

**Published:** 2026-04-08

**Authors:** Baorong Gao, Yali Miao, Hui Ye, Rui Miao

**Affiliations:** 1 Department of Obstetrics and Gynaecology, West China Second University Hospital, Chengdu 610041, China; 2 Key Laboratory of Birth Defects and Related Diseases of Women and Children (Sichuan University), Ministry of Education, No. 20, Section 3, Renmin Nan Lu, Chengdu 610041, China; 3 Graduate School of Zhejiang University, No. 866, Yuhangtang Road, Hangzhou 310058, China

**Keywords:** acute colonic pseudo-obstruction, cesarean section, factors, meta-analysis

## Abstract

**Objective**: Acute colonic pseudo-obstruction (ACPO), also known as Ogilvie syndrome, is a rare but serious complication following cesarean section (CS). Identifying factors associated with its occurrence is critical for early recognition and prevention. This systematic review and meta-analysis aimed to synthesize available evidence on factors associated with ACPO following CS. **Methods**: We performed a systematic literature search across five databases (PubMed, Embase, CNKI, Wanfang, and CBM) from inception to December 2025. Studies investigating factors associated with ACPO after CS were eligible. Quality of included studies was assessed using the Newcastle–Ottawa Scale. For factors reported in at least two studies, pooled odds ratios (ORs) or weighted mean differences (WMDs) with 95% confidence intervals (CIs) were calculated. **Results**: Five case-control studies comprising 484 patients (103 ACPO cases and 381 controls) were included, of which four were rated as good quality. Twenty-five potential associated factors were analyzed. Several pre-/intraoperative factors demonstrated statistically significant associations with ACPO risk, including concomitant anemia (OR = 8.94, 95% CI: 2.59–30.88), previous abdominal surgery (OR = 2.39, 95% CI: 1.28–4.47), surgery duration > 1 h (OR = 4.11, 95% CI: 2.20–7.67), and blood loss > 1000 mL (OR = 5.72, 95% CI: 2.10–15.58). Intraoperative blood loss as a continuous variable (WMD = 1.30, 95% CI: 0.14–2.46) was also significantly associated with ACPO. In contrast, emergency cesarean section, opioid use, and type of anesthesia were not significantly associated. Regarding postoperative features, bed rest > 12 h (OR = 2.66, 95% CI: 1.29–5.49), postoperative fever ≥ 38 °C (OR = 3.82, 95% CI: 1.94–7.54), elevated postoperative white blood cell count (WMD = 1.22, 95% CI: 0.30–2.14), and lower postoperative hemoglobin level (WMD = −0.50, 95% CI: −0.83 to −0.18) were significantly associated with ACPO. However, these factors may represent consequences of perioperative complications or components of the early clinical presentation of ACPO. **Conclusions**: This systematic review and meta-analysis identified multiple perioperative factors associated with ACPO following CS. However, the use of univariate data from a limited number of studies limits interpretability. Prospective cohort studies are needed to clarify whether these factors play a causal role in the development of ACPO.

## 1. Introduction

Acute colonic pseudo-obstruction (ACPO), or Ogilvie syndrome, is characterized by acute, massive dilation of the colon in the absence of mechanical obstruction [1]. It occurs most frequently in women following cesarean section (CS) [2] and can result in significant morbidity, including life-threatening complications such as colonic ischemia and perforation [3]. Timely diagnosis and prompt intervention—including electrolyte correction, fluid resuscitation, and, when necessary, surgical measures—can prevent disease progression in approximately 77% of cases [4]. However, diagnosis in postpartum ACPO is often delayed due to reduced peritoneal sensitivity from overstretched abdominal musculature [5]. Consequently, identifying associated risk factors is critical for early recognition, timely management, and improved prognosis.

Existing attempts to identify risk factors for ACPO after CS have relied primarily on retrospective designs, which often report inconsistent or contradictory findings [6,7,8,9,10]. These studies are further limited by small sample sizes, which may underpower the detection of significant associations. A prior systematic review by Jayaram et al. suggested that postpartum ACPO may be associated with emergency delivery, pre-eclampsia or HELLP syndrome (hemolysis, elevated liver enzymes, and low platelets), multiple pregnancy, antepartum hemorrhage, and placenta previa [11]. However, a quantitative synthesis was not possible due to the predominance of case reports and uncontrolled case series among the included studies. To address these evidence gaps, we conducted a systematic review and meta-analysis to synthesize the available data on risk factors for ACPO following CS.

## 2. Methods

This systematic review was conducted according to a prospectively registered protocol (PROSPERO CRD registration number: CRD4202451288100) [12] and adhered to the PRISMA (Preferred Reporting Items for Systematic Reviews and Meta-Analyses) (Supplementary Materials Table S1) and MOOSE (Meta-analysis Of Observational Studies in Epidemiology) guidelines [13,14].

### 2.1. Search Strategy

An initial comprehensive literature search was conducted from database inception to 18 December 2025, across PubMed, Embase (via Ovid), CNKI, Wanfang Database, and CBM. To ensure the inclusion of the most recent evidence, the search was updated just prior to journal submission. An experienced librarian (BG) developed the search strategy, employing the terms: (Ogilvie’s syndrome OR pseud obstruction OR pseudo-obstruction OR acute colonic pseud obstruction) AND (cesarean section OR caesarean section). No restrictions were placed on language or geography. We additionally hand-searched the reference lists of relevant systematic reviews to identify further studies.

### 2.2. Data Extraction and Risk of Bias Assessment

Two reviewers (RM and YM) independently performed data extraction using a predefined form, collecting details on study design, sample size, population, outcome definitions, and investigated risk factors. Disagreements were resolved through discussion until consensus was reached involving all authors. Corresponding authors of primary studies were contacted for clarification or if additional data as required.

The methodological quality of included studies was independently assessed by two reviewers (RM and YM) using the Newcastle–Ottawa Scale (NOS) [15]. Studies were classified as having a low (scores 7–9), intermediate (scores 4–6), or high (scores < 4) risk of bias.

### 2.3. Statistical Analysis

All analyses were performed using Stata software, version 12.0. For continuous variables (e.g., age, BMI), pooled estimates were calculated using a fixed-effect model when heterogeneity was low (*I*^2^ < 50%); otherwise, a random-effects model was applied. For dichotomous outcomes, pooled odds ratios (ORs) with 95% confidence intervals (CIs) were derived using the inverse variance method under either fixed- or random-effects models, as appropriate. A risk factor was eligible for meta-analysis only if reported in at least two studies. Heterogeneity was assessed using the *I*^2^ statistic and the between-study variance estimate (τ^2^). A two-sided *p*-value < 0.05 defined statistical significance.

Given the limited number of studies available for each risk factor and the presence of substantial heterogeneity, neither subgroup nor sensitivity analyses were feasible. Publication bias was not formally assessed due to the inclusion of fewer than ten studies per analysis.

## 3. Results

### 3.1. Identification of Studies

**Figure 1:**
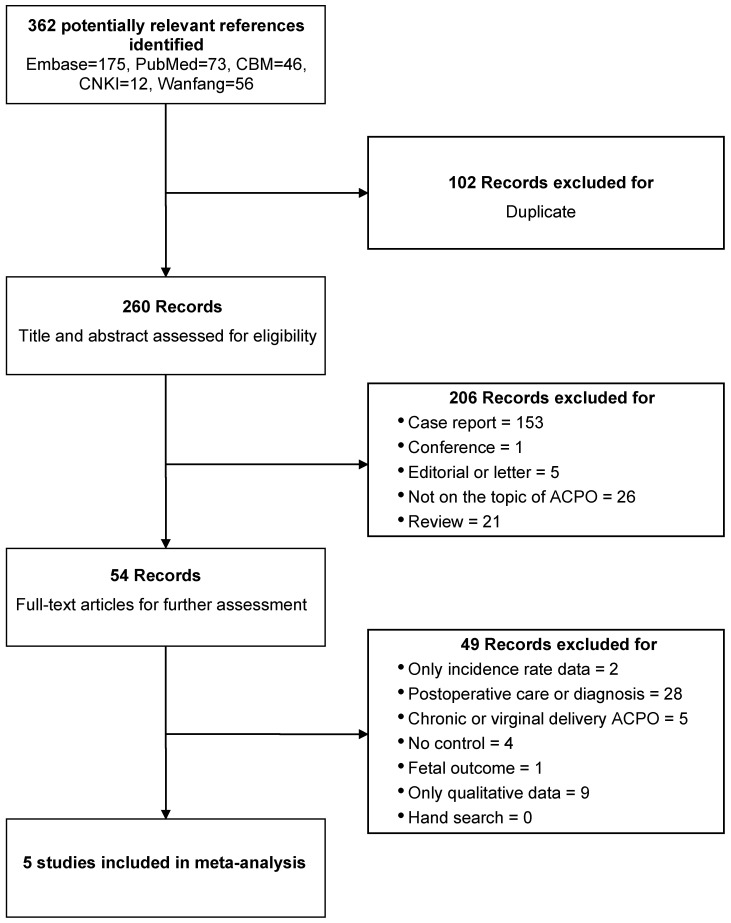
Flow diagram displaying all stages of the systematic review.

The systematic literature search yielded 362 records. After removing 102 duplicates, 260 articles underwent title and abstract screening. Following the exclusion of case reports, editorials, letters, conference abstracts, and reviews, 54 studies were selected for full-text review. Upon detailed evaluation, five studies met the predefined eligibility criteria and were included in the final meta-analysis (Figure 1).

### 3.2. Characteristics and Quality Assessment of Studies

The five included studies were all single-center case-control designs, encompassing 484 patients treated between 2005 and 2022. This total comprised 103 patients in the ACPO group (Definitions of ACPO in each study are provided in Supplementary Materials Table S2) and 381 controls. Three studies were conducted in China, one in Denmark and one in Australia. Study characteristics are summarized in Table 1. Based on the Newcastle–Ottawa Scale, four studies (80%) were rated as good quality and one (20%) as fair; detailed quality assessments are presented in Table 2.

### 3.3. Meta-Analysis of Factors Associated with ACPO After CS

This meta-analysis evaluated 25 factors associated with ACPO after CS, which were categorized into two groups: (i) candidate pre-/intraoperative risk factors; (ii) postoperative variables (Table 3). Other factors (e.g., fetal complication, ASA score) were reported in only a single study and were excluded from quantitative synthesis.

Continuous variables analyzed included age, BMI, preoperative potassium, preoperative white blood cell count, preoperative hemoglobin, preoperative albumin, gestational age, postoperative white blood cell (WBC) count, and postoperative hemoglobin. Estimated blood loss was analyzed as both a continuous and a dichotomous variable, because Christensen et al. [7] and Ford et al. reported blood loss as continuous data, while Peng et al. [6], Zhu et al. [8] and Wei et al. [9] reported it as a dichotomous variable (cut-off: 1000 mL). Other factors were analyzed as dichotomous variables. Pooled estimates are presented in Table 4. Heterogeneity, measured by the *I*^2^ statistic, ranged from 0% to 88.7%.

### 3.4. Candidate Pre-/Intraoperative Risk Factors

Two factors in this category demonstrated a statistically significant association with ACPO: concomitant anemia (OR = 8.94, 95% CI: 2.59–30.88, *p* = 0.001) and previous abdominal surgery (OR = 2.39, 95% CI: 1.28–4.47, *p* = 0.006). No significant associations were found for age, BMI, gestational diabetes, gestational hypertension, scarred uterus, and primiparity.

None of the preoperative variables analyzed—including preoperative potassium, WBC count, hemoglobin, albumin, premature rupture of membranes, singleton pregnancy, and gestational age—were significantly associated with ACPO risk.

Several intraoperative factors were identified as significant risk factors. Intraoperative blood loss exceeding 1000 mL was associated with increased risk for ACPO (OR = 5.72, 95% CI: 2.10–15.58, *p* = 0.001). When analyzed as a continuous variable, greater blood loss remained significant (WMD = 1.30, 95% CI: 0.14–2.46, *p* = 0.028). Surgery duration longer than one hour significantly increased risk for ACPO (OR = 4.11, 95% CI: 2.20–7.67, *p* < 0.001). Factors such as emergency cesarean section, opioid administration, and type of anesthesia were not significant.

### 3.5. Associated Postoperative Features

Prolonged postoperative bed rest (>12 h) (OR = 2.66, 95% CI: 1.29–5.49, *p* = 0.008) and postoperative fever (≥38 °C) (OR = 3.82, 95% CI: 1.94–7.54, *p* < 0.001) were significantly associated with ACPO. A higher postoperative white blood cell count was also significantly associated with ACPO (WMD = 1.22, 95% CI: 0.30–2.14, *p* = 0.010), while a lower postoperative hemoglobin level showed a significant inverse association (WMD = −0.50, 95% CI: −0.83 to −0.18, *p* = 0.003). However, these postoperative factors may reflect perioperative complications or early clinical features of ACPO rather than independent risk factors. The use of patient-controlled intravenous analgesia was not a significant factor.

## 4. Discussion

This systematic review synthesizes evidence from five case-control studies investigating factors associated with ACPO following cesarean delivery. Meta-analysis identified several factors associated with this serious complication: concomitant anemia, history of abdominal surgery, increased intraoperative blood loss, prolonged operative time, delayed postoperative ambulation, postoperative fever, and elevated postoperative WBC count. While the consistency of these factors supports the characterization of ACPO as a multifactorial disorder, causal inference cannot be established due to the observational designs and small sample sizes of the included studies.

The pathophysiology of ACPO remains incompletely understood, with the prevailing hypothesis centering on an imbalance in autonomic regulation of colonic motility [16]. Factors identified in this analysis can be integrated into this framework through several mediating mechanisms.

First, substantial intraoperative blood loss (and resulting anemia) and prolonged operative time may reduce splanchnic perfusion, leading to localized intestinal ischemia. Such ischemia can disrupt the function of the interstitial cells of Cajal and the enteric nervous system, which are essential for coordinating peristalsis [17]. Additionally, anemia and hemorrhage can activate a systemic stress response, increasing sympathetic outflow—a known inhibitor of gastrointestinal motility [18]. This provides a plausible link between anemia, high blood loss, and ACPO risk.

Second, the strong association with postoperative fever and leukocytosis underscores the role of inflammation. Surgical trauma, intra-abdominal contamination, or underlying infection can release pro-inflammatory cytokines [19], which directly inhibit colonic smooth muscle contractility and disrupt neuromuscular transmission [20]. These changes may promote the functional obstruction and dilatation characteristic of ACPO. A history of abdominal surgery may further increase risk due to pre-existing adhesions or neurovascular alterations that predispose the colon to dysmotility under the new stress of cesarean delivery [21]. Notably, postoperative fever and leukocytosis may be complications of cesarean section or early manifestations of ACPO, a distinction that requires further evaluation in future studies. Nonetheless, clinical attention to these symptoms is warranted.

Third, early mobilization is thought to stimulate parasympathetic activity and enhance gastrointestinal recovery [22], whereas delayed ambulation (>12 h) and prolonged recumbency may exacerbate colonic atony and gas accumulation, creating conditions favorable for pseudo-obstruction. However, delayed ambulation may also be a consequence rather than a cause of ACPO, requiring careful assessment of the clinical manifestations at diagnosis.

While individual studies proposed links involving opioid use or neuraxial anesthesia [10], our pooled analysis did not confirm these as independent risk factors, as adjusted odds ratios were not consistently reported across the original studies, precluding a meta-analysis of adjusted estimates. This finding suggests that their effect may be contingent on other coexisting conditions or be less pronounced than the factors identified here. However, as evidence supporting enhanced recovery after surgery (ERAS) protocols for cesarean delivery continues to accumulate [23], integrating factors such as opioid use or neuraxial anesthesia with demographic, surgical, and inflammatory markers may offer a more comprehensive framework for understanding the multifactorial origin of ACPO in postpartum patients, thereby improving risk assessment and guiding prevention.

Several limitations should be considered. First, the included studies each involved a limited number of ACPO cases while assessing multiple risk factors, increasing the potential for chance findings. Larger studies or individual patient data meta-analyses are needed to generate more precise estimates. Second, all studies used hospital-based case-control designs, permitting only the pooling of univariate risk estimates. This approach offers a lower level of evidence for causal inference compared with prospective cohort studies. These pooled univariate risk estimates may introduce bias due to potential confounding factors, such as the interplay between blood loss and anemia. Therefore, future studies using multivariable analyses or prospective cohort designs are needed. Third, detailed reporting was often lacking, making quality assessment less precise. Fourth, only five studies met the inclusion criteria, which is insufficient to explore the sources of heterogeneity or to assess publication bias. Therefore, the results of this study should be interpreted with caution.

## 5. Conclusions

In conclusion, this meta-analysis identified several perioperative factors associated with ACPO following cesarean delivery, rather than confirming causal factors, given that the dependence among these factors is likely high due to study limitations. Future prospective research in larger cohorts is needed to validate these associations and clarify their temporal interactions.

## Figures and Tables

**Table 1:** Characteristics of acute colonic pseudo-obstruction (ACPO) following cesarean section in the selected studies.

Study (Author, Year)	Design	Country	Inclusion Period	Cases (*n*)	Controls (*n*)	Case & Control Definitions	Factors Associated with ACPO
Peng et al. [6], 2019	1:5 case-control	China	2006–2018	11	55	Cases: Patients with post-cesarean ACPO. Controls: Patients without ACPO within 2 weeks as controls.	Age, BMI, pelvic adhesions, previous abdominopelvic surgery, emergency cesarean section, epidural anesthesia, preoperative serum potassium, preoperative albumin, preoperative WBC, preoperative hemoglobin, postoperative WBC, postoperative hemoglobin, intraoperative blood loss > 1000 mL, postoperative serum potassium, postoperative albumin, operative duration < 1 h, time to first ambulation > 24 h, use of patient-controlled analgesia pump, postoperative fever > 38 °C.
Christensen et al. [7], 2020	case-control	Denmark	2005–2016	25	37	Cases: Women who developed ACPO after cesarean section. Controls: Women who did not develop ACPO after cesarean section.	Age, BMI, gestational age, indications (fetal complication, maternal complications, or both), ASA score, parity, twins, cesarean section grade, syntocinon, morphine, anesthesia (general or regional), bleeding.
Zhu et al. [8], 2022	1:5 case-control	China	2018–2020	33	165	Cases: Patients who underwent emergency cesarean section complicated by ACPO. Controls: Patients who underwent emergency cesarean section without ACPO.	Age, BMI, gestational weeks, gestational hypertension, preoperative/postoperative WBC, hemoglobin, albumin, serum potassium, gestational diabetes, hypothyroidism, premature rupture of membranes, preoperative constipation, history of pelvic surgery, epidural labor analgesia, intraoperative blood loss > 1000 mL, operative duration > 1 h, intraoperative myomectomy, pelvic inflammatory disease found during operation, intraoperative use of protective drape, postoperative fever > 38 °C, time to first ambulation > 12 h.
Wei et al. [9], 2023	1:5 case-control	China	2018–2022	11	55	Cases: Patients with post-cesarean ACPO. Controls: Patients without post-cesarean ACPO within one-week post-cesarean.	Age, BMI, gestational weeks, gravidity, parity, number of fetuses, previous abdominopelvic surgery, use of patient-controlled analgesia pump, operative duration, scarred uterus, gestational hypertension, gestational diabetes, concomitant anemia, concomitant other surgery, premature rupture of membranes, reproductive system malformation, postpartum blood loss > 1000 mL, anesthesia type, timing of surgery (emergency or elective).
Ford et al. [10], 2023	1:3 case-control	Australia	2008–2016	23	69	Cases: Women with CT-diagnosed ACPO after cesarean delivery. Controls: Women without ACPO after cesarean delivery.	Maternal history (previous abdominal surgery, previous cesarean section), fetal characteristics (twins, breech, birthweight), diabetes, hypertensive disorder, antepartum hemorrhage, labor characteristics (diagnosis of labor, duration, oxytocin augmentation, opioid administration), intrapartum fever > 38 °C, emergency cesarean, anesthesia model (spinal, epidural, or general), estimated blood loss, postpartum hemorrhage.

BMI, body mass index; ASA, American Society of Anaesthesiologists; WBC, white blood cell; ACPO, acute colonic pseudo-obstruction.

**Table 2:** Quality assessment of included studies using the Newcastle–Ottawa Scale (NOS).

Studies	Selection				Comparability	Exposure			Overall
Author, Year	Is the Case Definition Adequate?	Representativeness of the Cases	Selection of Controls	Definition of Controls	Comparability of Cases and Controls	Ascertainment of Exposure	Same Methods of Ascertainment for Cases and Controls	Non-Response Rate	
Peng et al. [6], 2019	★	★	★	★	★★	★	★	☆	8/9
Christensen et al. [7], 2020	★	★	★	☆	★	★	★	☆	6/9
Zhu et al. [8], 2022	★	★	★	★	★	★	★	☆	7/9
Wei et al. [9], 2023	★	★	★	★	★	★	★	☆	7/9
Ford et al. [10], 2023	★	★	★	★	★★	★	★	☆	8/9

★ equals 1 point, ★★ equals 2 points, ☆ equals 0 point, with a total of 9 points.

**Table 3:** Data of investigated factors for ACPO following cesarean section by individual study.

Factor	Peng et al. [6]	Christensen et al. [7]	Zhu et al. [8]	Wei et al. [9]	Ford et al. [10]
Candidate pre-/intraoperative risk factors					
Age (years)	34.2 ± 5.6 vs. 31.6 ± 4.4	32.8 ± 4.6 vs. 32.7 ± 4.3	29.3 ± 3.8 vs. 30.4 ± 4.6	32.5 ± 3.8 vs. 31.2 ± 4.3	34.6 ± 4.5 vs. 34.0 ± 4.3
BMI	31.9 ± 6.3 vs. 30.6 ± 1.7	30.1 ± 7.0 vs. 29.7 ± 4.5	29.3 ± 4.6 vs. 29.3 ± 3.8	22.4 ± 3.3 vs. 24.7 ± 4.7	N/A
Gestational diabetes	N/A	N/A	9/33 vs. 36/165	4/11 vs. 11/55	3/22 vs. 10/63
Gestational hypertension	N/A	N/A	7/33 vs. 21/165	2/11 vs. 5/55	3/22 vs. 4/66
Concomitant anemia	N/A	N/A	N/A	5/11 vs. 3/55	3/22 vs. 2/64
Previous abdominal surgery	6/11 vs. 19/55	N/A	4/33 vs. 10/165	10/11 vs. 18/55	14/23 vs. 33/69
Scarred uterus	N/A	N/A	N/A	4/11 vs. 15/55	9/23 vs. 31/56
Primiparous	N/A	16/25 vs. 20/37	N/A	7/11 vs. 38/55	10/23 vs. 23/69
Preoperative potassium (mmol/L)	3.7 ± 0.2 vs. 3.7 ± 0.2	N/A	4.1 ± 0.4 vs. 3.9 ± 0.2	N/A	N/A
Preoperative WBC (×10^9^/L)	12.0 ± 1.7 vs. 11.1 ± 2.4	N/A	10.1 ± 2.2 vs. 9.8 ± 1.6	N/A	N/A
Preoperative hemoglobin (g/L)	104.9 ± 15.0 vs. 104.9 ± 9.8	N/A	112.3 ± 15.5 vs. 116.4 ± 10.1	N/A	N/A
Premature rupture of membranes	N/A	N/A	9/33 vs. 31/165	2/11 vs. 6/55	N/A
Preoperative albumin (g/L)	29.8 ± 2.5 vs. 31.7 ± 2.2	N/A	31.3 ± 4.0 vs. 31.3 ± 3.0	N/A	N/A
Singleton	N/A	23/25 vs. 33/37	N/A	8/11 vs. 55/55	21/23 vs. 68/69
Gestational age (weeks)	N/A	39.6 ± 0.7 vs. 39.2 ± 0.9	38.2 ± 2.1 vs. 38.2 ± 1.4	38.2 ± 0.9 vs. 38.9 ± 1.1	38.0 ± 2.7 vs. 38.0 ± 2.7
Emergency cesarean section	4/11 vs. 13/55	22/25 vs. 32/34		6/11 vs. 40/55	14/23 vs. 34/69
Opioid administration	N/A	19/19 vs. 32/34	N/A	N/A	4/8 vs. 4/19
Regional anesthesia	10/11 vs. 51/55	17/25 vs. 33/37	N/A	11/11 vs. 54/55	21/23 vs. 60/66
Blood loss (mL)	2/11 vs. 13/55 (>1000 mL)	662.6 ± 210.0 vs. 400.0 ± 47.1	5/33 vs. 7/165 (>1000 mL)	2/11 vs. 1/55 (>1000 mL)	634.8 ± 346.2 vs. 434.8 ± 248.6
Surgery duration > 1 h	4/11 vs. 9/55	N/A	22/33 vs. 51/165	8/11 vs. 20/53	N/A
Postoperative variables					
Postoperative bed rest > 12 h	3/11 vs. 4/55	N/A	23/33 vs. 82/165	N/A	N/A
Postoperative fever ≥ 38 °C	7/11 vs. 10/55	N/A	7/33 vs. 12/165	N/A	6/22 vs. 8/64
Patient-controlled analgesia pump	10/11 vs. 53/55	N/A	N/A	8/11 vs. 43/55	N/A
Postoperative WBC (×10^9^/L)	18.7 ± 4.4 vs. 11.9 ± 3.8	N/A	16.7 ± 4.6 vs. 13.6 ± 3.7	N/A	N/A
Postoperative hemoglobin (g/L)	87.6 ± 11.7 vs. 93.7 ± 9.0	N/A	101.2 ± 15.7 vs. 107.0 ± 12.0	N/A	N/A

Data are presented as mean ± standard deviation or n/n. BMI, Body mass index; WBC, White blood cell; N/A, not available.

**Table 4:** Pooled estimates for factors associated with ACPO after CS.

Factor	No. of Studies	Pooled Estimate (95% CI)	Statistical Method	Heterogeneity *I*^2^ (%)	*p*
Candidate pre-/intra-operative risk factors					
Age (years)	5	0.04 (−0.18–0.27)	WMD (IV, FEM)	28.9%	0.700
BMI	4	0.00 (−0.25–0.25)	WMD (IV, FEM)	28.6%	0.986
Gestational diabetes	3	1.36 (0.71–2.59)	OR (M-H, fixed)	0.0%	0.349
Gestational hypertension	3	2.03 (0.97–4.26)	OR (M-H, fixed)	0.0%	0.062
Concomitant anemia	2	8.94 (2.59–30.88)	OR (M-H, fixed)	0.0%	0.001
Previous abdominal surgery	4	2.39 (1.28–4.47)	OR (M-H, fixed)	32.4%	0.006
Scarred uterus	2	0.75 (0.34–1.68)	OR (M-H, fixed)	36.4%	0.485
Primiparous	3	1.32 (0.71–2.48)	OR (M-H, fixed)	0.00%	0.382
Preoperative potassium (mmol/L)	2	0.37 (−0.24–0.98)	WMD (IV, REM)	63.2%	0.230
Preoperative WBC (×10^9^/L)	2	0.20 (−0.13–0.52)	WMD (IV, FEM)	0.0%	0.233
Preoperative hemoglobin (g/L)	2	−0.28 (−0.602–0.047)	WMD (IV, FEM)	0.0%	0.094
Premature rupture of membranes	2	1.66 (0.77–3.59)	OR (M-H, fixed)	0.0%	0.200
Preoperative albumin (g/L)	2	−0.38 (−1.20–0.44)	WMD (IV, REM)	78.7%	0.367
Singleton	3	0.22 (0.02–2.30)	OR (M-H, random)	66.2%	0.203
Gestational age (weeks)	4	0.01 (−0.40–0.43)	WMD (IV, REM)	64.7%	0.959
Emergency cesarean section	4	1.14 (0.61–2.12)	OR (M-H, fixed)	0.0%	0.684
Opioid administration	2	3.55 (0.76–16.49)	OR (M-H, fixed)	0.0%	0.106
Regional anesthesia	4	0.50 (0.20–1.25)	OR (M-H, fixed)	0.0%	0.136
Blood loss (>1000 mL)	3	5.72 (2.10–15.58)	OR (M-H, fixed)	0.0%	0.001
Blood loss (mL)	2	1.30 (0.14–2.46)	WMD (IV, REM)	88.7%	0.028
Surgery duration > 1 h	3	4.11 (2.20–7.67)	OR (M-H, fixed)	0.0%	0.000
Postoperative variables					
Postoperative bed rest > 12 h	2	2.66 (1.29–5.49)	OR (M-H, fixed)	0.0%	0.008
Postoperative fever ≥ 38 °C	3	3.82 (1.94–7.54)	OR (M-H, fixed)	0.0%	0.000
Patient-controlled analgesia pump	2	0.62 (0.18–2.22)	OR (M-H, fixed)	0.0%	0.467
Postoperative WBC (×10^9^/L)	2	1.22 (0.30–2.14)	WMD (IV, REM)	80.9%	0.010
Postoperative hemoglobin (g/L)	2	−0.50 (−0.83 to −0.18)	WMD (IV, FEM)	0.0%	0.003

CI, confidence interval; WMD, weighted mean difference; OR, odds ratio; IV, inverse variance; FEM, fixed-effects model; REM, random-effects model; M-H, Mantel–Haenszel; WBC, white blood cell.

## Data Availability

No new data were created or analyzed in this study.

## Supplementary Materials

The following supporting information can be downloaded at https://www.mdpi.com/article/10.3390/jcm15082817/s1, Table S1: PRISMA_2020_checklist; Table S2: Diagnostic criteria for ACPO, multivariable-adjusted independent risk factors and covariates after cesarean delivery.

## Abbreviations

ACPO	Acute colonic pseudo-obstruction
CS	cesarean section
OR	odds ratio
WMD	weighted mean difference
CI	confidence interval
HELLP syndrome	hemolysis, elevated liver enzymes, and low platelets
PRISMA	Preferred Reporting Items for Systematic Reviews and Meta-Analyses
MOOSE	Meta-analysis of Observational Studies in Epidemiology
NOS	Newcastle–Ottawa Scale
ASA	American Society of Anaesthesiologists
BMI	Body mass index
WBC	White blood cell
